# The importance of strigolactone transport regulation for symbiotic signaling and shoot branching

**DOI:** 10.1007/s00425-016-2503-9

**Published:** 2016-04-04

**Authors:** Lorenzo Borghi, Guo-Wei Liu, Aurélia Emonet, Tobias Kretzschmar, Enrico Martinoia

**Affiliations:** Institute of Plant and Microbial Biology, University of Zurich, 8008 Zurich, Switzerland; Faculté de biologie et médecine, Département de biologie moléculaire végétale, Université de Lausanne, 1015 Lausanne, Switzerland; International Rice Research Institute (IRRI), Plant Breeding Genetics and Biotechnology, 4031 Laguna, Philippines

**Keywords:** Auxin, Cell-to-cell transport, Long-distance transport, PDR1, Phytohormone, Plant architecture, Polar localization, Transporter

## Abstract

**This review presents the role of strigolactone transport in regulating plant root and shoot architecture, plant-fungal symbiosis and the crosstalk with several phytohormone pathways. The authors, based on their data and recently published results, suggest that long-distance, as well local strigolactone transport might occur in a cell-to-cell manner rather than via the xylem stream.**

Strigolactones (SLs) are recently characterized carotenoid-derived phytohormones. They play multiple roles in plant architecture and, once exuded from roots to soil, in plant-rhizosphere interactions. Above ground SLs regulate plant developmental processes, such as lateral bud outgrowth, internode elongation and stem secondary growth. Below ground, SLs are involved in lateral root initiation, main root elongation and the establishment of the plant-fungal symbiosis known as mycorrhiza. Much has been discovered on players and patterns of SL biosynthesis and signaling and shown to be largely conserved among different plant species, however little is known about SL distribution in plants and its transport from the root to the soil. At present, the only characterized SL transporters are the ABCG protein PLEIOTROPIC DRUG RESISTANCE 1 from *Petunia axillaris* (PDR1) and, in less detail, its close homologue from *Nicotiana tabacum* PLEIOTROPIC DRUG RESISTANCE 6 (PDR6). PDR1 is a plasma membrane-localized SL cellular exporter, expressed in root cortex and shoot axils. Its expression level is regulated by its own substrate, but also by the phytohormone auxin, soil nutrient conditions (mainly phosphate availability) and mycorrhization levels. Hence, PDR1 integrates information from nutrient availability and hormonal signaling, thus synchronizing plant growth with nutrient uptake. In this review we discuss the effects of PDR1 de-regulation on plant development and mycorrhization, the possible cross-talk between SLs and other phytohormone transporters and finally the need for SL transporters in different plant species.

## Introduction

Strigolactones (SLs) are carotenoid derived phytohormones involved in several plant developmental processes. Initially discovered as germination stimulants for parasitic weeds (Cook et al. 1966), it is now established that SLs also induce the initial steps of the plant-fungal symbiosis mycorrhiza (Akiyama et al. 2005) and regulate plant root and shoot architecture (reviewed in Al-Babili and Bouwmeester 2015). SLs act as integrators of plant growth with nutrient availability in soil: mainly low phosphate and/or nitrogen conditions induce SL biosynthesis transport and root-to-soil exudation from the starving plants (Yoneyama et al. 2007; Lopez-Raez and Bouwmeester 2008; Foo et al. 2013). As consequence, increased SL concentrations inhibit lateral bud outgrowth, induce lateral root initiation and initiate root colonization by arbuscular mycorrhizal fungi. The latter strategy enables the plant to increase nutrient uptake by expanding the plant root system with the extended fungal hyphal system.

The multiple roles and targets of SLs suggest that SL biosynthesis and transport are strongly interconnected and are fine-tuned at multiple levels, to enable swift and appropriate response to diverse inner and outer stimuli. SL biosynthesis has been extensively investigated in different plant species (reviewed in Lopez-Obando et al. 2015). These investigations showed that SLs are synthetized by shared players: one iron containing protein (DWARF27) expressed in the root and shoot vasculature (Lin et al. 2009; Waters et al. 2012), two CAROTENOID CLEAVAGE DIOXYGENASE/MORE AXILLARY GROWTH (CCD7/MAX3 and CCD8/MAX4) expressed in roots and shoots (Sorefan et al. 2003; Booker et al. 2005) and finally a plant-species specific number of cytochrome P450 monoxygenases (MAX1 and MAX1-like), expressed in root and shoot vasculatures (Challis et al. 2013; Zhang et al. 2014b). Although the players involved in SL biosynthesis are conserved, their activities can greatly vary between plant species. For example, canonical SLs such as orobanchol and deoxystrigol were characterized as main regulators of plant development and root-to-soil interactions in *Oryza sativa* (Xie et al. 2013), *Petunia hybrida* (Kretzschmar et al. 2012) and *Solanum lycopersicum* (Lopez-Raez et al. 2008). A different situation is present in *Arabidopsis*, where acid derivatives of carlactone, a biologically active SL precursor, were reported to be present in the xylem and able to inhibit shoot lateral branching (Abe et al. 2014; Seto et al. 2014). Also, the expression pattern of the several enzymes involved in SL biosynthesis is not yet fully characterized except for *Arabidopsis* (Sorefan et al. 2003; Booker et al. 2004, 2005; Shen et al. 2007). Grafting experiments revealed that SLs are synthetized in both plant shoots and roots (Domagalska and Leyser 2011). Nevertheless, wild-type root stocks are able to complement SL biosynthesis mutant scions, thus suggesting that a long distance transport of SL from the root to the shoot happens, possibly to integrate the regulation of shoot growth with the nutrient availability perceived by the root.

SL signaling is also conserved among different plant species. The heterodimeric receptor for SL consists of the F-Box protein MAX2 and the alpha/beta hydrolase DWARF14 (D14). D14 hydrolyzes the SL molecule at the enol-ether bond, between the SL tricyclic lactone (ABC ring) and the butenolide moiety (D ring). SL signal transduction is then carried on by the D14-D ring complex, which promotes the ubiquitination and subsequent proteolysis of DWARF53 (D53), suggesting that D53 is a repressor of SL signaling. D53 interacts in vitro with TOPLESS (TPL) and TPL-related co-repressors (Jiang et al. 2013; Zhou et al. 2013), which possibly regulate downstream transcription factors such as *FINE CULM1/BRANCHED1 (FC1/BRC1)* (Minakuchi et al. 2010; Braun et al. 2012), involved in SL-induced repression of lateral bud outgrowth. Interestingly, the D14-D ring complex targets also the gibberellin *SIGNALING REPRESSOR 1 (SLR1)* (Nakamura et al. 2013), and the brassinosteroid signaling factors *BRASSINOSTEROID INSENSITIVE EMS SUPPRESSOR 1 (BES1)* and *BRASSINAZOLE 1 (BZR1)* (Wang et al. 2013) supporting the existence of crosstalk between SL, gibberellin and brassinosteroid signaling pathways.

MAX2 can also form a heterodimeric receptor with the D14 paralogue KARRIKIN INSENSITIVE 2 (KAI2). This receptor is capable of detecting karrikins (KARs), bioactive components from smoke that are stimulants for germination (Flematti et al. 2004, 2009; van Staden et al. 2004), and a yet unknown hypothesized KAI2 plant endogenous ligand (KL) (Conn and Nelson 2016). Although KARs (and possibly KL) and SLs have different effects on plant development, they share MAX2 and they are assumed to start a similar downstream cascade of events: KARs regulate specific aspects of plant development such as seed germination, seedling growth and leaf development (Stanga et al. 2013; Bennett and Leyser 2014; Soundappan et al. 2015). Interestingly, MAX2 (and therefore possibly both KARs and SLs signaling pathways) is downregulated by sucrose (Barbier et al. 2015), thus suggesting a sucrose-KARs-SLs network in control of plant development.

Despite their importance, still little is known about the pathways involved in SL transport within the plant and from the root to the soil, as well as about their regulation and synchronization with SL biosynthesis and soil nutrient availability. Up to date the only characterized SL transporter is the ATP BINDING CASSETTE (ABC) protein PDR1 from petunia (Kretzschmar et al. 2012). Its putative ortholog in *Nicotiana tabacum* PDR6 (Xie et al. 2015a) indicates that SL transporters are conserved in Solanaceae, while in *Arabidopsis thaliana* the sequence homologue AtABCG40 is a reported abscisic acid (ABA) transporter (Kang et al. 2010). The G-type ABC (PDR) transporters are known to play an important role in phytohormone transport, such as for cytokinins (CKs), ABA, auxin derivatives like indole butyric acid (IBA) and SLs (reviewed in Borghi et al. 2015). However, sequence homology between ABCG proteins is not as informative about the transported substrate, like in the above mentioned *Arabidopsis*/petunia case. Also, the high duplication levels of PDR1 homologues make the isolation and characterization of new SL transporters in plant species other than petunia not easy. Last but not least, among the few known SL targets there are transporters of the phytohormone auxin, such as PIN-FORMED1 and PIN-FORMED2 (PIN1 and PIN2) (reviewed in Adamowski and Friml 2015). PIN1 was reported to be quickly depleted from the plasma membrane by SL exogenous applications (Shinohara et al. 2013), while PIN2 localization in the plasma membrane and its endocytic recycling was shown to be regulated by SL affecting the cytoskeleton dynamics (Pandya-Kumar et al. 2014). Therefore, especially reverse genetic approaches to isolate new SL transporters are hindered by the difficulty to analyze phenotypes due to SL crosstalk with auxin transporters.

In this review the state-of-the-art of SL transport in roots and shoots is reported, with focus on the need for SL transporters to regulate the distribution and tissue-specific fine-tuning of this phytohormone. Furthermore new PDR1 homologue candidates to expand the investigation of SL transport in new plant species are proposed.

## Transport of SL in roots

A first indirect indication that SLs are exported from the root to the soil was provided by the observation that SLs induce the germination of parasitic weeds (Cook et al. 1966; Matusova et al. 2005). The further discovery that hyphal branching of arbuscular mycorrhizal fungi (AMF) is induced by SLs suggested that SL export from roots is a vital process for the plant to compete in environments with sparingly available phosphate resources (Akiyama et al. 2005). Grafting experiments, gene expression patterns and localization experiments have shown that SL biosynthesis occurs in roots as well as in shoot tissues such as stem and fruits (Domagalska and Leyser 2011; Lopez-Obando et al. 2015). However, these experiments also provided evidence that roots from wild type plants could supply SLs—or a SL precursor, as *max1* mutant root stocks can still recover *max3* or *max4* mutant scions (Booker et al. 2005)—to the shoot, implying root-to-shoot directed transport. Such shoot-ward SL transport might be necessary to equilibrate SL levels between the shoot and the root in order that the whole plant architecture synchronizes its growth depending on nutrient conditions, e.g., low phosphate, which inhibits shoot lateral bud outgrowth while concomitantly inducing lateral root development. In summary, these observations indicated that SL transport within the root occurs in two directions, from the root to the soil to induce hyphal branching and promote mycorrhization as well as from the root to the shoot to sustain SL-mediated processes in the aerial part of the plant. Using a targeted approach Kretzschmar and colleagues (Kretzschmar et al. 2012) identified PDR1 in roots of petunia. Evidence that PDR1 is indeed a SL transporter came from the observations that *pdr1* mutant plants excreted only minor amounts of SLs and consequently were mycorrhized much less efficiently than wild type. The mycorrhization levels of *pdr1* mutants are nearly as low as in the SL biosynthesis mutant *decreased apical dominance 1 (dad1)*, thus suggesting that PDR1 is the main player in SL root exudation. Furthermore, *Arabidopsis* plants overexpressing *PDR1* became tolerant against high SL concentrations in the growth medium, suggesting that their capacity to excrete SLs was strongly increased.

**Fig. 1:**
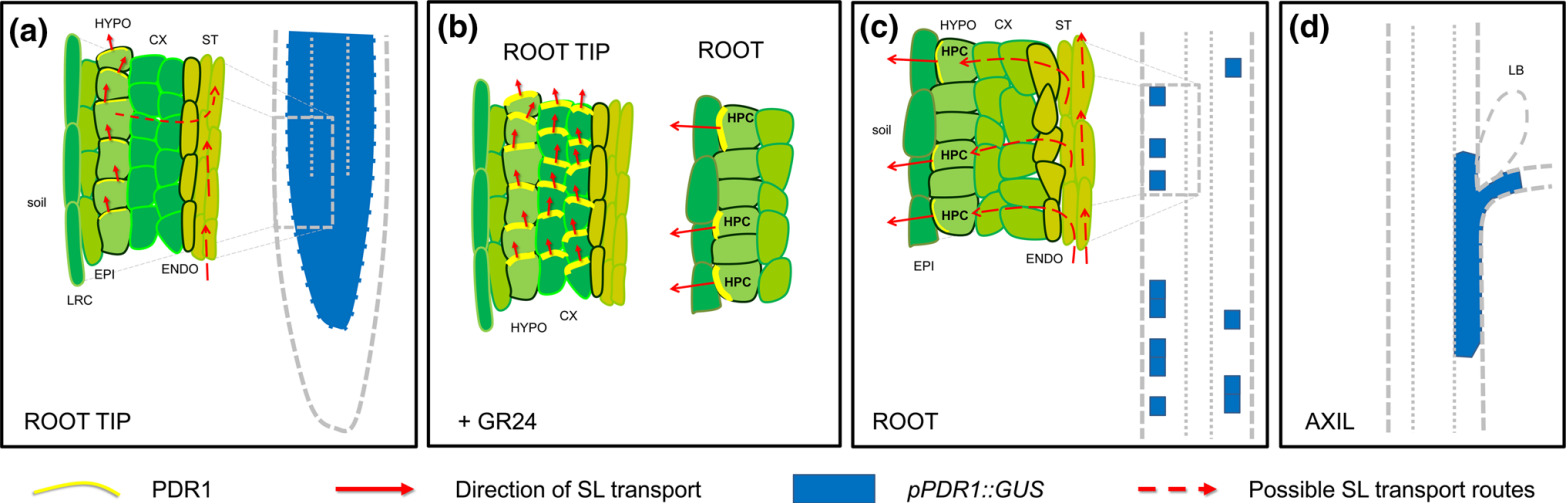
Routes of SL transport based on *pPDR1::GUS* activity and GFP-PDR1 detection. *Dashed red arrows* represent possible SL routes based on SL detection in the xylem sap of tomato (Kohlen et al. 2012). **a** *pPDR1::GUS* is expressed in the root tip but absent from lateral root cap (LRC) and epidermis (EPI, unpublished data). PDR1 protein is apically localized in the hypodermal cells (HYPO). **b** After GR24 treatment, PDR1 protein is also present in the deeper cortex layers (CX) of the root tip, but not visible in the endodermis or stele (ENDO and ST). **c** Above the root tip, *pPDR1::GUS* is present in hypodermal passage cells (HPCs). In HPCs, PDR1 is outer-laterally localized. PDR1 protein levels are boosted in HPC by GR24 treatments (**b**). **d** *pPDR1::GUS* is expressed in cells subtending the shoot lateral axils, close but excluded from the dormant lateral bud (LB)

In this first report PDR1 was shown to be localized in the root tip and in hypodermal passage cells via *pPDR1::GUS*. Hypodermal passage cells (HPCs) are non-suberized cells located in the hypodermis that serve as entry points for the mycorrhizal fungus: their distribution along the root affects mycorrhization success (Sharda and Koide 2008). In subsequent experiments Sasse and colleagues (Sasse et al. 2015) analyzed the localization of the protein fusion GFP-PDR1 to get a more detailed expression pattern and/or subcellular localization of PDR1. The authors could show that PDR1 co-localizes with CCD8/DECREASED APICAL DOMINANCE 1 (DAD1) in the root tip, where in *Arabidopsis thaliana* CCD8/MAX4 was also detected (Sorefan et al. 2003). Interestingly, PDR1 was asymmetrically localized in the plasma membrane of root-tip cortical cells, and co-localized with the auxin efflux facilitator PIN2 in the cortex cells that expressed both transporters. PIN2 is apically localized in the cells of this root region: previous investigations on auxin transport showed that the apical localization of PIN2 in epidermis and cortex is responsible for a shoot-ward, polar transport of the hormone auxin from the root apex upwards (Wisniewska et al. 2006). Based on PDR1 and PIN2 co-localization in the root tip cortex cells, the authors suggested that the function of PDR1 in the root tip is loading the synthesized SL into the apoplast of basal tissues, i.e., either the vasculature or the root cortex (Fig. 1a). *pdr1* mutants are indeed more prone to the accumulation of exogenously applied GR24 (a synthetic SL) in the root tip and show alterations in root tip homeostasis, such as cell division. Furthermore, the strong down-regulation of *CDD8*/*DAD1* observed in *pdr1* root tips indicates that SL biosynthesis is feedback regulated by its own substrate. Such negative feedback might be also useful to avoid SL accumulation in the root tip that could be detrimental by inducing hyphal branching of mycorrhizal fungi towards the wrong root side: root tips have no suberized hypodermis and potentially SL could diffuse out of the root tip in the rhizosphere, thus inducing hyphal branching and penetration in the dividing root meristem. Additionally, PDR1 protein levels are increased by exogenous GR24 treatments: such observation is in line with the need for the root tip to remove SLs accumulating close to the root meristem. Interestingly, GR24 not only increases PDR1 protein amounts but also expands PDR1 protein pattern to deeper cortical layers (Fig. 1b): it is still unclear whether GR24 just induces *PDR1* expression levels or also increases the stability of PDR1 protein. Besides the root tip, the PDR1 promoter (*pPDR1*) is furthermore active in cortical cells along the vasculature, but excluded from the stele. In this differentiated region of the root, HPCs are present. Analysis of its sub-cellular localization revealed PDR1 to be confined to the outer-lateral plasma membrane of HPCs, consistent with its role in exuding SLs towards the rhizosphere to facilitate mycorrhization (Fig. 1c). These results show that within the same organ PDR1 exhibits a dual polar localization. A missing link, however, is how shoot-ward transported SLs are delivered to HPCs.

The observation that PDR1 exhibits an asymmetrical localization in petunia root tips indicates that at least in this region of the root active cell-to-cell transport occurs. It is therefore important to identify in the future the transporter(s) responsible for cellular uptake of SL in order to understand the entire cell-to-cell flux of SL. An active cell-to-cell transport hypothesis is supported by recent work using fluorescent-tagged SL (Fridlender et al. 2015). The authors showed that after disrupting ATP-dependent processes SL influx increased while SL efflux decreased, suggesting that SL importer(s) and exporter(s) are involved in the regulation of SL cell-to-cell flux.

**Fig. 2:**
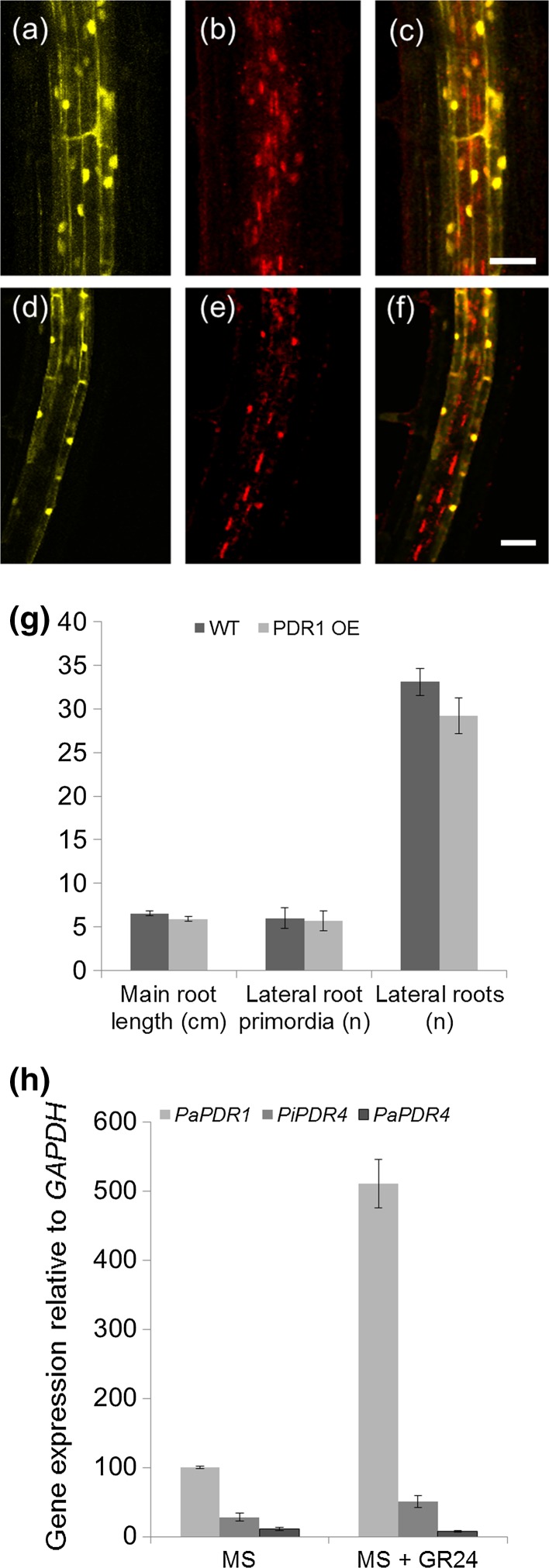
**a**–**f** *pPDR1::nls*-*YFP* (**a**, **d**) and *pPIN1::nls*-*RFP* (**b**, **e**) nuclear localized signals (nls) and merged (c, f) in *Arabidopsis* root cortex: **a**–**c** cortical view, **d**–**f** vasculature view. *pPDR1* is active in cells surrounding the *pPIN1* domain and outside of the stele. **g** Col-0 (wild type); *DR5::GUS* and PDR1 OE; *DR5::GUS* *Arabidopsis* roots do not show significant morphological/GUS pattern differences, e.g., for main root length, lateral root primordia formation and lateral root number (number of lines analyzed = 12; seedlings screened per line = 5; *cm* centimeters; *n* number). **h** Expression levels of *PDR1* compared to *PaPDR4* and *Petunia inflata* *PDR4* (*PiPDR4*) ± exogenous GR24 treatment (± s.e.m. of 3 biological replicates). *Scale bars* = 50 µm

Interestingly, *Arabidopsis* plants expressing the *pPDR1* fusion with YFP do not show any signal in vascular cells, where *pPIN1::RFP* is visible (Fig. 2a–f), an observation that is also made for the protein fusion GFP-PDR1 in Petunia (Sasse et al. 2015). These results support the hypothesis that in petunia SL transport might occur via PDR1 in root cortical layers, and not or not only via the xylem, as initially suggested for *Arabidopsis* and *Solanum lycopersicum* but then supported only for the latter (Andreo-Jimenez et al. 2015). Our analyses, focusing on *PDR1* overexpression (PDR1 OE) in *Arabidopsis*, showed that PDR1 can transport and exude radiolabeled GR24 even in a plant species that is not related to petunia. However, we did not observe PDR1 OE effects on *Arabidopsis* plant architecture (Fig. 2g). By contrast, a clear phenotype was observed in petunia lines overexpressing *PDR1* (Sasse et al. 2015), suggesting that PDR1 specifically transports orobanchol (the most abundant SL in petunia), GR24 and possibly other SL derivatives, but not carlactone or methyl carlactone, the most abundant SL-like bioactive molecules in *Arabidopsis* (Abe et al. 2014). The fact that no shoot branching phenotype or other SL-related phenotypes have been identified in a large screen of *Arabidopsis* ABCG transporter mutants (unpublished data) could indicate that carlactone or methyl carlactone are transported by multiple and redundant ABC transporters or by other transporters not related to ABC transporters, making the reverse genetic approach less effective. For abscisic acid it has been shown that, besides ABCG transporters, NITRATE TRANSPORTERS (NRTs) and MULTI MICROBIAL EXTRUSION PROTEINS (MATEs) can catalyze the transfer through membranes (Boursiac et al. 2013).

*PDR1* was unexpectedly found to be upregulated after mycorrhization (Kretzschmar et al. 2012), when SL exudation was reported to be reduced (Lendzemo et al. 2007). However, expression analyses showed that at this stage the transporter was still induced within the root cortex. We therefore suggest that SL transport regulation might exhibit also a guidance function in the already colonized root, via induction of intraradical hyphal branching after initial mycorrhiza establishment. In support of this hypothesis, intraradical hyphae and arbuscules were reported to be induced in *dad1* mutants, but not in WT plants, after GR24 treatments (Breuillin et al. 2010), while the number of established contacts with the roots (hyphopodia) was not altered by this treatment. Similar investigations are necessary on *pdr1* mutants to test if exogenous GR24 is as effective as in *dad1* for recovering the mutant, or if the SL transport and/or distribution via PDR1 are necessary for the intra-root hyphal elongation.

Although SL transport in petunia seems to rely largely on PDR1 there might be multiple, additional SL transporters in other plant species. Promising SL transporter candidates should demonstrate induction by low nutrient conditions and/or exogenous GR24 and should be located in the plasma membrane of root cells. A variety of SL transporters is possibly required for the allocation of specific amounts of different SLs in plants like *Oryza sativa*, where precise SL blends were shown to either affect AMF hyphal branching or parasitic weed germination (Jamil et al. 2011). By contrast, specific SLs are reported to stimulate AMF hyphal branching, while some others induced germination of parasitic weeds (Akiyama et al. 2010). At the moment PDR1 is the only characterized SL transporter: it will be important to identify its main substrate and its orthologs from other species to better understand how plants can organize hormonal signaling that can be either benign or detrimental to plant growth.

## Transport of SL in shoots

Previous grafting experiments in *Arabidopsis* with wild-type plants and different *max* (SL-deficient) mutants not only showed that SLs and/or SL precursors are transported from the root to the shoot but also demonstrated that shoot-synthesized SLs are sufficient to support above ground SL functions, when no SL is translocated shoot-wards (Domagalska and Leyser 2011). However, to date little is known about SL transport within the shoot and so far it is unclear whether it occurs only over short- or also across long-distance, i.e., only from lateral axils to dormant buds or, e.g., also between internodes.

In the shoot PDR1 has been localized close to lateral axils (Fig. 1d), while it is absent in dormant buds (Kretzschmar et al. 2012). Both *pdr1* and the *Nicotiana tabacumpdr6* mutant, the latter deficient in the *PDR1* homolog *NtPDR6*, lose control over shoot lateral branching inhibition and show earlier bud outgrowth compared to the corresponding wild type. As expected, plants overexpressing *PDR1* are impaired in bud outgrowth, suggesting that ectopic SL transport towards the axillary buds is sufficient to delay shoot lateral development (Sasse et al. 2015). However, as SL biosynthesis also occurs close to lateral buds (Umehara et al. 2008; Mashiguchi et al. 2009), it is not clear yet whether PDR1-related branching phenotypes are caused by deregulated root-to-shoot SL transport or by local feedback regulation of SL biosynthesis.

In shoots like in roots, SL biosynthetic tissues are spread along the vasculature or localized in specific organs. *MAX1* and *CCD8/MAX4* are expressed in vascular and aerial parts of *Arabidopsis* (Bainbridge et al. 2005; Mashiguchi et al. 2009). The tomato *MAX3*-homolog *SlCCD7* was detected in stems but also in immature tomato fruits (Vogel et al. 2010). These expression patterns support the hypothesis that there might be two different routes of SL transport in the shoot: one to distribute locally synthetized SL to adjacent tissues (e.g., from axils to buds) and one to transport SL across a long distance, possibly via the vasculature, for instance to regulate leaf senescence (Yamada et al. 2014; Ueda and Kusaba 2015). A detailed site-map of SL synthesis, transport and transporters is still necessary to support this hypothesis. Orobanchol and two additional SL-like compounds were previously detected in the xylem sap of *Arabidopsis* and they were suggested to regulate shoot architecture responses under phosphate-limiting conditions (Kohlen et al. 2011). Later research showed, however, that the main players in *Arabidopsis* shoot lateral branching inhibition are not canonical SLs but the carlactone methyl ester derivative of carlactonic acid. Besides, no SLs were detected in *Arabidopsis* xylem sap (Abe et al. 2014; Seto et al. 2014; Xie et al. 2015b). Nevertheless, old and recent grafting studies with wild type and SL-like biosynthesis mutants (*max1, max3,* and *max4*) plants showed that wild type root stocks can suppress the branching phenotype of mutant scions, indicating that also SL precursors like methyl carlactone can be transported from roots to shoots (Sorefan et al. 2003; Teichmann and Muhr 2015).

SLs are involved in the regulation of different plant developmental processes, often in cross-talk with other phytohormones such as auxins (Al-Babili and Bouwmeester 2015). Two models have been proposed to account for the regulation of shoot lateral branching operated by auxins and SLs. First, experimental evidence supports that SL dampens polar auxin transport in the main stem, suggesting that SLs can influence bud outgrowth by down-regulating the auxin efflux facilitator PIN1 in the plasma membrane, hence inhibiting auxin canalization out of dormant buds (Shinohara et al. 2013). The second model proposes local action of SLs as second messengers of auxin transported into buds, where they induce the expression of the TCP-family transcription factor *BRC1,* an inhibitor of bud outgrowth (Braun et al. 2012; Dun et al. 2013; Lauressergues et al. 2015). The mechanisms behind these divergent or co-existing views have not been fully elucidated, and investigations on auxin and SL transport fluxes and patterns are still ongoing. Previous studies in petunia and *Pisum sativum* showed that both the SL transporter *PDR1* and the SL biosynthesis genes *CCD7/RAMOSUS5 (RMS5)* and *CCD8/RAMOSUS1 (RMS1)* are upregulated by auxin (Hayward et al. 2009; Kretzschmar et al. 2012). If auxins induce SL transport and synthesis but on the other side SL inhibits auxin transport, we have to hypothesize that additional regulatory signals are necessary to inhibit SL transport or boost auxin transport to maintain auxin canalization out of the dormant bud. Recently, Mason and colleagues (Mason et al. 2014) demonstrated that plants regulate axillary bud outgrowth by controlling the amount of sugar translocated to the shoot axils. Therefore, sucrose transport likely is part of the regulation of lateral bud outgrowth, which is supported by recent findings for *Sorghum bicolor* (Kebrom and Brutnell 2015). Sucrose was shown to induce the expression of the auxin efflux carrier gene *PIN1* in *Rosa hybrida* and *pPIN:PIN1*-*GFP* fluorescence abundance in the plasma membrane of *Solanum lycopersicum* (Barbier et al. 2015). Hence, sucrose seems to be the primary regulator of lateral bud outgrowth, providing carbon and inducing auxin transport and canalization. Apart from this, the direct targets and downstream pathways of sucrose signaling that affect bud release have not been fully elucidated. Exogenous applied sucrose promotes bud outgrowth in non-decapitated plants and down-regulates *BRC1* expression within the first 2 h of incubation in *Pisum sativum* (Mason et al. 2014). *MAX2* was recently shown to be downregulated by sucrose in *Rosa hybrida* (Barbier et al. 2015). Ongoing studies might reveal if SL transport and/or biosynthesis are further targets of sucrose signaling and therefore play a sugar-dependent role on bud growth release.

In *Pisum sativum*, CKs and SLs were reported, respectively, to negatively and positively regulate the expression of *PsBRC1* in dormant buds (Braun et al. 2012). As long as *PsBRC1* expression levels are high, bud outgrowth is abolished. It has been suggested that both SLs and CKs regulate *PsBRC1* transcript levels through the signaling perceived by the SL receptor MAX2/D14 (Janssen et al. 2014). Besides this signaling crosstalk, little is known about possible feedback regulations between transporters of CKs and SLs. The CK transporter ABCG14 was described as being essential for root to shoot translocation of trans-Zeatin (tZ)-cytokinins (Ko et al. 2014; Zhang et al. 2014a), but its expression pattern in buds or internodes is not yet known. Recently, gibberellins (GA) were also reported to interact with SL signaling and to regulate shoot branching in rice and *Jatropha curcas* (Nakamura et al. 2013; Ni et al. 2015). However, at present the knowledge on GA transporters is restricted to flower organs (Saito et al. 2015). Likewise, brassinosteroids were found to be involved in the SL-mediated regulation of shoot branching through the brassinosteroid signaling suppressors *BES1* (Wang et al. 2013; Waldie et al. 2014). Nevertheless, the mechanisms underlying brassinosteroid transport are still unknown.

## The need for SL transporters

Up to now, SL transporters have been isolated only from Solanaceae: PDR1 from petunia (Kretzschmar et al. 2012) and a very close homolog, PDR6 from *Nicotiana tabacum* (Xie et al. 2015a). For the latter, transport experiments have not been carried out, however, similar to PDR1, NtPDR6 regulates shoot lateral branching and is expressed in root tips, root cortex cells and shoot lateral axils. At present, no SL transporter has been isolated from monocotyledonous species or in the model plant *Arabidopsis*, where the closest sequence homolog of PDR1 is the ABA transporter AtABCG40 (Kang et al. 2010).

**Fig. 3:**
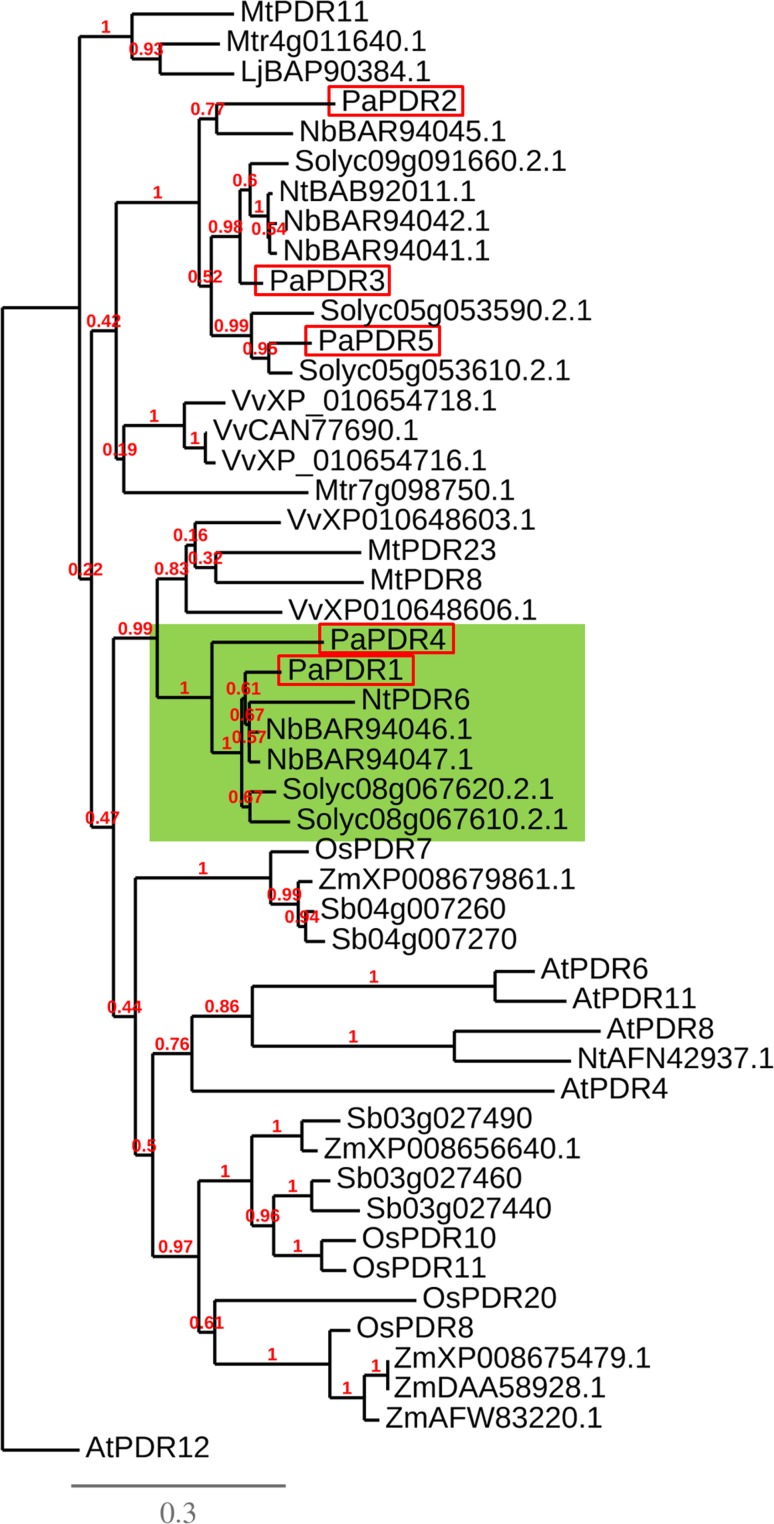
Phylogenetic tree of PDR1 protein sequence homologs of *Petunia axillaris (Pa), Nicotiana tabacum (Nt), Nicotiana benthamiana (Nb), Solanum lycopersicum (Sl), Medicago truncatula (Mt), Lotus japonicas (Lj), Vitis vinifera (Vv), Oryza sativa (Os),* *Sorghum bicolor (Sb), Arabidopsis thaliana (At) and Zea mays (Zm).* Not yet investigated, closely related sequences in the same plant species might be representatives of effective gene duplications or not yet complete genome curation. PaPDR1 and petunia homologs are red squared; the PaPDR1 clade is highlighted in green. Phylogenetic tree (bootstraps: 100) created via Phylogeny.fr (Dereeper et al. 2008)

When carrying out PDR1 phylogeny analyses on mono- and di-cotyledons to identify SL transporters in other plant families, possible duplication events for PDR1 homologues in *Solanum lycopersicum*, *Medicago truncatula*, *Vitis vinifera, Oryza sativa*, *Sorghum bicolor*, *Zea mays* and *Nicotiana benthamiana* were detected (Fig. 3). In *Petunia axillaris* PLEIOTROPIC DRUG RESISTANCE 4 (PDR4) is the closest homolog to PDR1. PDR4, however, is not strongly expressed in roots and not induced by GR24 (Fig. 3h), and hence is unlikely to contribute significantly to SL transport. Thus, sequence homologies within ABCG coding sequences are not a reliable indicator for identifying the transporter’s substrates. Duplications might also be present in monocots such as *Oryza sativa*, *Sorghum bicolor* and *Zea mays* (Fig. 3). Therefore, the generation of loss-of-function lines for single and multiple genes is necessary. A possible interesting case seems to be *Lotus japonicus*, where only a single PDR1 homolog is present, making this plant attractive for studies on SL transport. However, the lotus genome is at present only 67 % sequenced and hence further homologs may be present in the yet unsequenced part.

## Conclusion

The recent investigations on PDR1 showed that not only biosynthesis and signal reception, but also transport of the phytohormone SL plays a main role in regulation of plant development and plant-fungal symbiosis. Although no PDR1 ortholog was characterized out of Solanaceae yet, the need for a SL transporter is probably widespread among different plant species. It was recently shown that no SLs were detected in the xylem sap of *Solanum lycopersicum*, *Oryza sativa*, *Nicotiana tabacum*, *Sorghum bicolor* and *Arabidopsis thaliana* (Xie et al. 2015b). Additionally, the authors showed that exogenous SLs added to the plant root reached the shoot only 20 h after treatment, thus suggesting active cell-to-cell routes of SL transport alternative to the faster xylem sap stream. The role of the SL cellular-exporter PDR1 in regulating this cell-to-cell transport and the search for SL cellular-importers are at the moment under investigation. The isolation of PDR1 orthologs in crops and staple food species to study SL transport and its effects on plant development will still take quite some time due to gene redundancy. As several traits induced by mis-expression of PDR1 and consequent mis-targeted SL transport are of agricultural interest, e.g., changes in shoot architecture and enhanced nutrient uptake via mycorrhiza induction, the expression of PDR1 in distantly related plant species could be an initial strategy to estimate the positive and negative effects on plant development and biomass production of enhanced SL transport.

### *Author contribution statement*

LB, GL and EM conceived and designed research. LB, AE and TK conducted experiments. LB, GL and EM wrote the manuscript. All authors read and approved the manuscript.

## Abbreviations

ABC: ATP binding cassette
BRC1: Branched 1
CKs: Cytokinins
KARs: Karrikins
MAX: More axillary growth
PDR: Pleiotropic drug resistance
SLs: Strigolactones
